# 5′-Amino-2-oxo-2′,3′-dihydro­spiro­[indoline-3,7′-thieno[3,2-*b*]pyran]-6′-carbonitrile 1′,1′-dioxide

**DOI:** 10.1107/S1600536810032861

**Published:** 2010-08-28

**Authors:** Shi-De Shen, Xiao-Dong Feng, Wei-Hua Yang, Chang-Sheng Yao

**Affiliations:** a Xuzhou Institute of Architectural Technology, Xuzhou 221116, People’s Republic of China; b School of Chemistry and Chemical Engineering, Xuzhou Normal University, Xuzhou 221116, People’s Republic of China; c Key Laboratory of Biotechnology for Medicinal Plants, Xuzhou Normal University, Xuzhou 221116, People’s Republic of China

## Abstract

In the title compound, C_15_H_11_N_3_O_4_S, the dihedral angle between the mean planes of the dihydro­indol-2-one (r.m.s. deviation = 0.015 Å) and dihydro­thieno[3,2-*b*]pyran (r.m.s. deviation = 0.011 Å) ring systems is 89.53 (3)°. The crytal packing is consolidated by inter­molecular N—H⋯O and N—H⋯N hydrogen bonds, which link the mol­ecules into a two-dimensional network into sheets lying parallel to (100).

## Related literature

For the anti­viral and α2-adrenoreceptor agonist activity of thieno[3,2-*b*]pyran derivatives, see: Chao *et al.* (2009 ▶); Friary *et al.* (1991 ▶). For the biological and pharmacological properties of indole derivatives, see: Sundberg (1996 ▶).
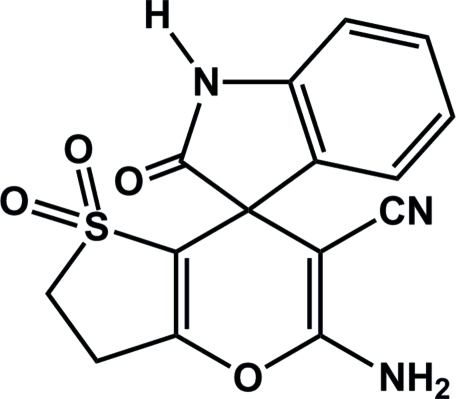


## Experimental

### 

#### Crystal data

C_15_H_11_N_3_O_4_S
*M* *_r_* = 329.33
Monoclinic, 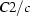

*a* = 30.669 (4) Å
*b* = 8.1760 (14) Å
*c* = 12.229 (2) Å
β = 112.611 (8)°
*V* = 2830.7 (8) Å^3^
*Z* = 8
Mo *K*α radiation
μ = 0.25 mm^−1^
*T* = 113 K
0.25 × 0.22 × 0.20 mm

#### Data collection

Rigaku Saturn724 CCD diffractometer
Absorption correction: multi-scan (*CrystalClear-SM Expert*; Rigaku/MSC, 2009 ▶) *T*_min_ = 0.939, *T*_max_ = 0.951
19659 measured reflections
4149 independent reflections
3670 reflections with *I* > 2σ(*I*)
*R*_int_ = 0.037

#### Refinement

*R*[*F* ^2^ > 2σ(*F* ^2^)] = 0.036
*wR*(*F* ^2^) = 0.099
*S* = 1.08
4149 reflections
220 parameters
H atoms treated by a mixture of independent and constrained refinement
Δρ_max_ = 0.44 e Å^−3^
Δρ_min_ = −0.27 e Å^−3^

### 

Data collection: *CrystalClear-SM Expert* (Rigaku/MSC, 2009 ▶); cell refinement: *CrystalClear-SM Expert*; data reduction: *CrystalClear-SM Expert*; program(s) used to solve structure: *SHELXS97* (Sheldrick, 2008 ▶); program(s) used to refine structure: *SHELXL97* (Sheldrick, 2008 ▶); molecular graphics: *SHELXTL* (Sheldrick, 2008 ▶); software used to prepare material for publication: *SHELXTL*.

## Supplementary Material

Crystal structure: contains datablocks I, global. DOI: 10.1107/S1600536810032861/ci5148sup1.cif

Structure factors: contains datablocks I. DOI: 10.1107/S1600536810032861/ci5148Isup2.hkl

Additional supplementary materials:  crystallographic information; 3D view; checkCIF report

## Figures and Tables

**Table 1:** Hydrogen-bond geometry (Å, °)

*D*—H⋯*A*	*D*—H	H⋯*A*	*D*⋯*A*	*D*—H⋯*A*
N1—H1⋯N3^i^	0.89 (2)	2.21 (2)	3.0878 (15)	170 (2)
N1—H2⋯O4^ii^	0.88 (2)	2.01 (2)	2.8790 (14)	172 (1)
N2—H3⋯O3^iii^	0.84 (2)	2.13 (2)	2.9255 (14)	159 (2)

Symmetry codes: (i) 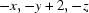; (ii) 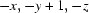; (iii) 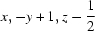.

**Fig. 1.**
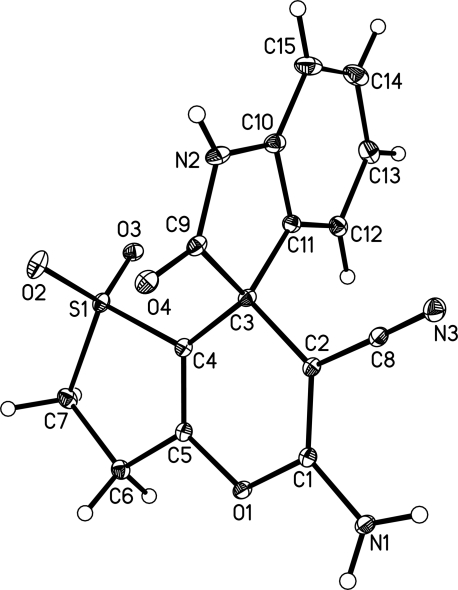
The molecular structure of the title compound, showing 30% probability displacement ellipsoids and the atom-numbering scheme.

**Fig. 2.**
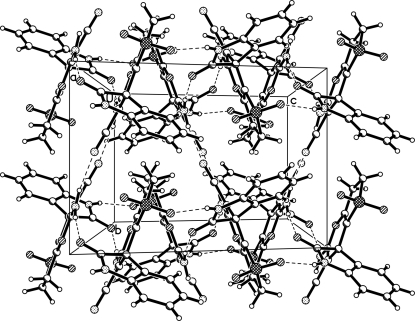
A packing diagram of the title compound. Intermolecular hydrogen bonds are shown as dashed lines.

## supplementary crystallographic information

### Comment

The indole core represents an interesting pharmacophore with the feature of biological and pharmacological properties (Sundberg, 1996). Thienopyranyl compounds, such as thieno[3,2-*b*]pyran derivatives, can be uesed as antiviral agents (Friary *et al.*,1991) and α-2 C adrenoreceptor agonists (Chao *et al.*, 2009). This led us to pay much attention to the synthesis and bioactivity of compounds containing these two significant fragments. During the synthesis of spiro[indoline-3,7'-thieno[3,2-*b*]pyran] derivatives, the title compound was isolated and its structure was determined by X-ray analysis. The results are presented here.

In the title molecule (Fig. 1), the dihydroindole-2-one ring system is planar (r.m.s. deviation 0.015 Å); the largest deviation from the mean plane is 0.023 (1) Å for atom C3. The dihydrothieno[3,2-*b*]pyran unit is also planar with an r.m.s. deviation of 0.011 Å (maximum deviation from the plane is 0.022 Å for atom C2). The dihedral angle between the two units is 89.53 (3)°.

The crystal packing is stabilized by intermolecular N—H···O and N—H···N hydrogen bonds (Table 1) which link the molecules into a two-dimensional network parallel to the (100) [Fig.2].

### Experimental

The title compound was synthesized by the reaction of dihydrothiophen-3(2*H*)-one-1,1-dioxide (1 mmol) and 2-(2-oxoindolin-3-ylidene)malononitrile (1 mmol) catalyzed by piperidine (0.02 g) in 10 ml ethanol under reluxing until completion (monitored by TLC). Cooling the reaction mixture slowly gave single crystals suitable for X-ray diffraction.

### Refinement

The H atoms bound to N atoms were located in a difference map and were refined freely [refined N–H lengths, 0.89 (2), 0.88 (2) and 0.84 (2) Å]. All other H atoms were placed in calculated positions, with C–H = 0.95, or 0.99 Å, and included in the final cycles of refinement using a riding model, with U_iso_(H) = 1.2U_eq_(parent atom).

### Crystal data

**Table:**

C_15_H_11_N_3_O_4_S	*F*(000) = 1360
*M**_r_* = 329.33	*D*_x_ = 1.546 Mg m^−^^3^
Monoclinic, *C*2/*c*	Mo *K*α radiation, λ = 0.71075 Å
Hall symbol: -C 2yc	Cell parameters from 6345 reflections
*a* = 30.669 (4) Å	θ = 1.4–33.4°
*b* = 8.1760 (14) Å	µ = 0.25 mm^−^^1^
*c* = 12.229 (2) Å	*T* = 113 K
β = 112.611 (8)°	Prism, colourless
*V* = 2830.7 (8) Å^3^	0.25 × 0.22 × 0.20 mm
*Z* = 8	

### Data collection

**Table:**

Rigaku Saturn724 CCD diffractometer	4149 independent reflections
Radiation source: rotating anode	3670 reflections with *I* > 2σ(*I*)
multilayer	*R*_int_ = 0.037
Detector resolution: 14.222 pixels mm^-1^	θ_max_ = 30.1°, θ_min_ = 1.4°
ω scans	*h* = −42→43
Absorption correction: multi-scan (*CrystalClear*-SM Expert; Rigaku/MSC, 2009)	*k* = −11→11
*T*_min_ = 0.939, *T*_max_ = 0.951	*l* = −17→17
19659 measured reflections	

### Refinement

**Table:**

Refinement on *F*^2^	Primary atom site location: structure-invariant direct methods
Least-squares matrix: full	Secondary atom site location: difference Fourier map
*R*[*F*^2^ > 2σ(*F*^2^)] = 0.036	Hydrogen site location: inferred from neighbouring sites
*wR*(*F*^2^) = 0.099	H atoms treated by a mixture of independent and constrained refinement
*S* = 1.08	*w* = 1/[σ^2^(*F*_o_^2^) + (0.0595*P*)^2^ + 0.5864*P*] where *P* = (*F*_o_^2^ + 2*F*_c_^2^)/3
4149 reflections	(Δ/σ)_max_ = 0.001
220 parameters	Δρ_max_ = 0.44 e Å^−^^3^
0 restraints	Δρ_min_ = −0.27 e Å^−^^3^

### Special details

**Table:**

Geometry. All e.s.d.'s (except the e.s.d. in the dihedral angle between two l.s. planes) are estimated using the full covariance matrix. The cell e.s.d.'s are taken into account individually in the estimation of e.s.d.'s in distances, angles and torsion angles; correlations between e.s.d.'s in cell parameters are only used when they are defined by crystal symmetry. An approximate (isotropic) treatment of cell e.s.d.'s is used for estimating e.s.d.'s involving l.s. planes.
Refinement. Refinement of *F*^2^ against ALL reflections. The weighted *R*-factor *wR* and goodness of fit *S* are based on *F*^2^, conventional *R*-factors *R* are based on *F*, with *F* set to zero for negative *F*^2^. The threshold expression of *F*^2^ > σ(*F*^2^) is used only for calculating *R*-factors(gt) *etc*. and is not relevant to the choice of reflections for refinement. *R*-factors based on *F*^2^ are statistically about twice as large as those based on *F*, and *R*- factors based on ALL data will be even larger.

### Fractional atomic coordinates and isotropic or equivalent isotropic displacement parameters (Å^2^)

**Table:**

	*x*	*y*	*z*	*U*_iso_*/*U*_eq_	
S1	0.147537 (9)	0.29057 (3)	0.24536 (2)	0.01647 (9)	
O1	0.02172 (3)	0.46422 (10)	0.16158 (7)	0.01616 (16)	
O2	0.16145 (3)	0.24351 (12)	0.15034 (8)	0.0273 (2)	
O3	0.18485 (3)	0.33938 (10)	0.35503 (7)	0.01955 (17)	
O4	0.10067 (3)	0.52484 (10)	−0.05002 (7)	0.02012 (18)	
N1	−0.02109 (3)	0.68754 (13)	0.09053 (9)	0.0183 (2)	
N2	0.16403 (3)	0.67827 (13)	0.06897 (9)	0.0202 (2)	
N3	0.05132 (3)	0.96181 (13)	0.00106 (9)	0.0214 (2)	
C1	0.02156 (4)	0.62030 (13)	0.12026 (9)	0.0145 (2)	
C2	0.06085 (4)	0.68905 (13)	0.11213 (9)	0.0145 (2)	
C3	0.10896 (3)	0.60632 (13)	0.15197 (9)	0.0142 (2)	
C4	0.10317 (4)	0.43896 (13)	0.19455 (9)	0.0143 (2)	
C5	0.06325 (4)	0.37984 (13)	0.19645 (9)	0.0145 (2)	
C6	0.06143 (4)	0.21074 (13)	0.23920 (10)	0.0180 (2)	
H6A	0.0382	0.1442	0.1757	0.022*	
H6B	0.0520	0.2125	0.3081	0.022*	
C7	0.11110 (4)	0.13877 (14)	0.27463 (11)	0.0209 (2)	
H7A	0.1100	0.0386	0.2282	0.025*	
H7B	0.1241	0.1098	0.3598	0.025*	
C8	0.05546 (4)	0.84058 (13)	0.05249 (9)	0.0159 (2)	
C9	0.12351 (4)	0.59395 (14)	0.04311 (9)	0.0164 (2)	
C10	0.18025 (4)	0.74686 (15)	0.18382 (10)	0.0191 (2)	
C11	0.14933 (4)	0.70594 (13)	0.23842 (10)	0.0160 (2)	
C12	0.15829 (4)	0.75477 (14)	0.35300 (10)	0.0192 (2)	
H12	0.1377	0.7253	0.3911	0.023*	
C13	0.19871 (4)	0.84902 (15)	0.41138 (11)	0.0246 (3)	
H13	0.2057	0.8843	0.4903	0.029*	
C14	0.22859 (4)	0.89130 (17)	0.35519 (12)	0.0290 (3)	
H14	0.2556	0.9564	0.3964	0.035*	
C15	0.22021 (4)	0.84121 (17)	0.24027 (12)	0.0278 (3)	
H15	0.2409	0.8702	0.2022	0.033*	
H1	−0.0266 (6)	0.792 (2)	0.0714 (15)	0.032 (4)*	
H2	−0.0445 (6)	0.623 (2)	0.0855 (14)	0.030 (4)*	
H3	0.1762 (6)	0.6867 (19)	0.0186 (15)	0.030 (4)*	

### Atomic displacement parameters (Å^2^)

**Table:**

	*U*^11^	*U*^22^	*U*^33^	*U*^12^	*U*^13^	*U*^23^
S1	0.01688 (14)	0.01640 (14)	0.01632 (15)	0.00426 (9)	0.00659 (11)	0.00220 (9)
O1	0.0148 (3)	0.0163 (4)	0.0191 (4)	0.0019 (3)	0.0083 (3)	0.0031 (3)
O2	0.0321 (5)	0.0310 (5)	0.0228 (4)	0.0128 (4)	0.0148 (4)	0.0019 (4)
O3	0.0151 (3)	0.0225 (4)	0.0195 (4)	0.0017 (3)	0.0049 (3)	0.0025 (3)
O4	0.0215 (4)	0.0236 (4)	0.0169 (4)	0.0021 (3)	0.0092 (3)	0.0017 (3)
N1	0.0147 (4)	0.0185 (5)	0.0227 (5)	0.0022 (4)	0.0083 (4)	0.0021 (4)
N2	0.0168 (4)	0.0264 (5)	0.0208 (5)	0.0008 (4)	0.0110 (4)	0.0044 (4)
N3	0.0212 (5)	0.0191 (5)	0.0243 (5)	0.0030 (4)	0.0092 (4)	0.0034 (4)
C1	0.0163 (4)	0.0159 (5)	0.0120 (5)	0.0013 (4)	0.0061 (4)	−0.0001 (4)
C2	0.0143 (5)	0.0152 (5)	0.0143 (5)	0.0024 (4)	0.0058 (4)	0.0012 (4)
C3	0.0128 (4)	0.0152 (5)	0.0151 (5)	0.0018 (4)	0.0057 (4)	0.0019 (4)
C4	0.0155 (5)	0.0148 (5)	0.0128 (5)	0.0022 (4)	0.0055 (4)	0.0021 (4)
C5	0.0163 (5)	0.0150 (5)	0.0125 (5)	0.0019 (4)	0.0057 (4)	0.0005 (4)
C6	0.0206 (5)	0.0158 (5)	0.0185 (5)	−0.0005 (4)	0.0085 (4)	0.0019 (4)
C7	0.0206 (5)	0.0150 (5)	0.0228 (6)	0.0002 (4)	0.0036 (4)	0.0034 (4)
C8	0.0137 (4)	0.0177 (5)	0.0165 (5)	0.0019 (4)	0.0060 (4)	−0.0012 (4)
C9	0.0167 (5)	0.0173 (5)	0.0171 (5)	0.0049 (4)	0.0088 (4)	0.0054 (4)
C10	0.0152 (5)	0.0200 (5)	0.0215 (6)	0.0022 (4)	0.0064 (4)	0.0050 (4)
C11	0.0131 (5)	0.0144 (5)	0.0186 (5)	0.0014 (3)	0.0040 (4)	0.0033 (4)
C12	0.0192 (5)	0.0163 (5)	0.0195 (5)	0.0017 (4)	0.0047 (4)	0.0023 (4)
C13	0.0216 (5)	0.0206 (6)	0.0231 (6)	0.0000 (4)	−0.0008 (5)	0.0007 (4)
C14	0.0179 (5)	0.0263 (6)	0.0339 (7)	−0.0048 (5)	0.0001 (5)	0.0032 (5)
C15	0.0157 (5)	0.0304 (7)	0.0344 (7)	−0.0034 (5)	0.0065 (5)	0.0075 (5)

### Geometric parameters (Å, °)

**Table:**

S1—O2	1.4359 (9)	C3—C9	1.5617 (15)
S1—O3	1.4448 (9)	C4—C5	1.3248 (14)
S1—C4	1.7491 (11)	C5—C6	1.4867 (15)
S1—C7	1.7957 (12)	C6—C7	1.5319 (16)
O1—C5	1.3648 (12)	C6—H6A	0.99
O1—C1	1.3719 (13)	C6—H6B	0.99
O4—C9	1.2221 (14)	C7—H7A	0.99
N1—C1	1.3340 (14)	C7—H7B	0.99
N1—H1	0.887 (17)	C10—C15	1.3869 (16)
N1—H2	0.877 (17)	C10—C11	1.3942 (16)
N2—C9	1.3480 (15)	C11—C12	1.3792 (16)
N2—C10	1.4133 (16)	C12—C13	1.3998 (16)
N2—H3	0.838 (18)	C12—H12	0.95
N3—C8	1.1543 (15)	C13—C14	1.3842 (19)
C1—C2	1.3673 (15)	C13—H13	0.95
C2—C8	1.4148 (15)	C14—C15	1.390 (2)
C2—C3	1.5228 (14)	C14—H14	0.95
C3—C4	1.4987 (14)	C15—H15	0.95
C3—C11	1.5190 (15)		
			
O2—S1—O3	116.71 (6)	C7—C6—H6A	110.4
O2—S1—C4	109.33 (5)	C5—C6—H6B	110.4
O3—S1—C4	111.62 (5)	C7—C6—H6B	110.4
O2—S1—C7	112.17 (6)	H6A—C6—H6B	108.6
O3—S1—C7	109.72 (5)	C6—C7—S1	107.79 (8)
C4—S1—C7	95.25 (5)	C6—C7—H7A	110.1
C5—O1—C1	117.12 (8)	S1—C7—H7A	110.1
C1—N1—H1	122.4 (11)	C6—C7—H7B	110.1
C1—N1—H2	117.5 (11)	S1—C7—H7B	110.1
H1—N1—H2	119.9 (15)	H7A—C7—H7B	108.5
C9—N2—C10	112.12 (10)	N3—C8—C2	177.90 (12)
C9—N2—H3	120.1 (11)	O4—C9—N2	126.97 (10)
C10—N2—H3	127.8 (11)	O4—C9—C3	125.10 (10)
N1—C1—C2	127.58 (10)	N2—C9—C3	107.86 (9)
N1—C1—O1	110.36 (9)	C15—C10—C11	121.93 (12)
C2—C1—O1	122.06 (9)	C15—C10—N2	128.42 (11)
C1—C2—C8	117.77 (9)	C11—C10—N2	109.66 (10)
C1—C2—C3	124.51 (10)	C12—C11—C10	120.70 (10)
C8—C2—C3	117.44 (9)	C12—C11—C3	130.77 (10)
C4—C3—C11	114.92 (9)	C10—C11—C3	108.54 (10)
C4—C3—C2	106.69 (8)	C11—C12—C13	117.97 (11)
C11—C3—C2	114.50 (9)	C11—C12—H12	121.0
C4—C3—C9	110.35 (9)	C13—C12—H12	121.0
C11—C3—C9	101.79 (8)	C14—C13—C12	120.66 (12)
C2—C3—C9	108.38 (8)	C14—C13—H13	119.7
C5—C4—C3	124.95 (9)	C12—C13—H13	119.7
C5—C4—S1	109.79 (8)	C13—C14—C15	121.88 (12)
C3—C4—S1	125.24 (8)	C13—C14—H14	119.1
C4—C5—O1	124.61 (10)	C15—C14—H14	119.1
C4—C5—C6	120.35 (10)	C10—C15—C14	116.85 (12)
O1—C5—C6	115.04 (9)	C10—C15—H15	121.6
C5—C6—C7	106.80 (9)	C14—C15—H15	121.6
C5—C6—H6A	110.4		
			
C5—O1—C1—N1	−179.52 (9)	C5—C6—C7—S1	−1.03 (11)
C5—O1—C1—C2	0.99 (15)	O2—S1—C7—C6	−112.07 (8)
N1—C1—C2—C8	−8.53 (18)	O3—S1—C7—C6	116.50 (8)
O1—C1—C2—C8	170.86 (9)	C4—S1—C7—C6	1.28 (9)
N1—C1—C2—C3	177.78 (10)	C10—N2—C9—O4	178.17 (11)
O1—C1—C2—C3	−2.82 (17)	C10—N2—C9—C3	0.86 (12)
C1—C2—C3—C4	2.39 (14)	C4—C3—C9—O4	58.53 (13)
C8—C2—C3—C4	−171.31 (9)	C11—C3—C9—O4	−179.02 (10)
C1—C2—C3—C11	−125.94 (11)	C2—C3—C9—O4	−57.96 (14)
C8—C2—C3—C11	60.36 (13)	C4—C3—C9—N2	−124.10 (10)
C1—C2—C3—C9	121.23 (11)	C11—C3—C9—N2	−1.65 (11)
C8—C2—C3—C9	−52.48 (12)	C2—C3—C9—N2	119.41 (10)
C11—C3—C4—C5	127.60 (11)	C9—N2—C10—C15	−179.69 (12)
C2—C3—C4—C5	−0.48 (15)	C9—N2—C10—C11	0.40 (13)
C9—C3—C4—C5	−118.03 (11)	C15—C10—C11—C12	−1.83 (17)
C11—C3—C4—S1	−54.22 (13)	N2—C10—C11—C12	178.09 (10)
C2—C3—C4—S1	177.70 (7)	C15—C10—C11—C3	178.57 (11)
C9—C3—C4—S1	60.16 (12)	N2—C10—C11—C3	−1.51 (12)
O2—S1—C4—C5	114.52 (9)	C4—C3—C11—C12	−58.40 (15)
O3—S1—C4—C5	−114.83 (8)	C2—C3—C11—C12	65.64 (15)
C7—S1—C4—C5	−1.19 (9)	C9—C3—C11—C12	−177.67 (11)
O2—S1—C4—C3	−63.89 (11)	C4—C3—C11—C10	121.14 (10)
O3—S1—C4—C3	66.76 (10)	C2—C3—C11—C10	−114.81 (10)
C7—S1—C4—C3	−179.61 (10)	C9—C3—C11—C10	1.88 (11)
C3—C4—C5—O1	−1.14 (18)	C10—C11—C12—C13	1.27 (16)
S1—C4—C5—O1	−179.56 (8)	C3—C11—C12—C13	−179.23 (11)
C3—C4—C5—C6	179.18 (10)	C11—C12—C13—C14	−0.03 (17)
S1—C4—C5—C6	0.76 (13)	C12—C13—C14—C15	−0.73 (19)
C1—O1—C5—C4	0.96 (15)	C11—C10—C15—C14	1.04 (18)
C1—O1—C5—C6	−179.33 (9)	N2—C10—C15—C14	−178.86 (12)
C4—C5—C6—C7	0.21 (14)	C13—C14—C15—C10	0.22 (19)
O1—C5—C6—C7	−179.51 (9)		

### Hydrogen-bond geometry (Å, °)

**Table:**

*D*—H···*A*	*D*—H	H···*A*	*D*···*A*	*D*—H···*A*
N1—H1···N3^i^	0.89 (2)	2.21 (2)	3.0878 (15)	170 (2)
N1—H2···O4^ii^	0.88 (2)	2.01 (2)	2.8790 (14)	172 (1)
N2—H3···O3^iii^	0.84 (2)	2.13 (2)	2.9255 (14)	159 (2)

Symmetry codes: (i) −*x*, −*y*+2, −*z*; (ii) −*x*, −*y*+1, −*z*; (iii) *x*, −*y*+1, *z*−1/2.
